# Multimodality Imaging for Right Ventricular Function Assessment in Severe Tricuspid Regurgitation

**DOI:** 10.3390/jcm13175076

**Published:** 2024-08-27

**Authors:** Francesco Melillo, Dario Fabiani, Alessandro Santoro, Pietro Oro, Francesca Frecentese, Luigi Salemme, Tullio Tesorio, Eustachio Agricola, Michele De Bonis, Roberto Lorusso

**Affiliations:** 1 Heart and Vascular Centre, Cardiovascular Research Institute, University of Maastricht, 6221 Maastricht, The Netherlands; francescomelillo1989@gmail.com (F.M.); roberto.lorussobs@gmail.com (R.L.); 2 Echo Lab, Clinica Montevergine GVM Care and Research, 83013 Mercogliano, Italy; dario.fabiani94@gmail.com (D.F.); pietrooro94@gmail.com (P.O.); francesca.frecentese92@gmail.com (F.F.); 3 Intensive Care Unit, Clinica Montevergine GVM Care and Research, 83013 Mercogliano, Italy; 4 Interventional Cardiology Unit, Clinica Montevergine GVM Care and Research, 83013 Mercogliano, Italy; ginosalemme@hotmail.it (L.S.); tulliotesorio@gmail.com (T.T.); 5 Heart Valve Centre, IRCCS Ospdeale San Raffaele, 20132 Milan, Italy; agricola.eustachio@hsr.it (E.A.); debonis.michele@hsr.it (M.D.B.)

**Keywords:** severe tricuspid regurgitation, right ventricle, tricuspid valve intervention, multimodality imaging, right ventricular dysfunction

## Abstract

Severe tricuspid regurgitation (TR) is a pathological condition associated with worse cardiovascular outcomes. In the vicious cycle of right ventricular compensation and maladaptation to TR, the development of right ventricle (RV) dysfunction has significant prognostic implications, especially in patients undergoing surgical or percutaneous treatments. Indeed, RV dysfunction is associated with increased operative morbidity and mortality in both surgical and percutaneously treated patients. In this context, the identification of clinical or subtle right ventricle dysfunction plays a critical role inpatient selection and timing of surgical or percutaneous tricuspid valve intervention. However, in the presence of severe TR, evaluation of RV function is challenging, given the increase in preload that may lead to an overestimation of systolic function for the Frank–Starling law, reduced reliability of pulmonary artery pressure estimation, the sensitivity of RV to afterload that may result in afterload mismatch after treatment. Consequently, conventional echocardiographic indices have some limitations, and the use of speckle tracking for right ventricular free wall longitudinal strain (RV-FWLS) analysis and the use of 3D echocardiography for RV volumes and ejection fraction estimation are showing promising data. Cardiac magnetic resonance (CMR) represents the gold standards for volumes and ejection fraction evaluation and may add further prognostic information. Finally, cardiac computer tomography (CCT) provides measurements of RV and annulus dimensions that are particularly useful in the transcatheter field. Identification of subtle RV dysfunction may need, therefore, more than one imaging technique, which will lead to tip the balance between medical therapy and early intervention towards the latter before disease progression. Therefore, the aim of this review is to describe the main imaging techniques, providing a comprehensive assessment of their role in RV function evaluation in the presence of severe TR.

## 1. Introduction

Studies on the natural history of patients with tricuspid regurgitation (TR) have highlighted the high disease prevalence and the worse outcomes associated with increasing TR severity. The estimated prevalence of all-cause TR was 0.55%, similar to the prevalence of aortic stenosis and approximately one-fourth of all left-sided valves disease. After years of debate, it is now established that isolated TR is also associated with decreased survival at up to 10 years of follow-up [1]. This led to increasing interest in understanding the pathology and seeking treatments. However, isolated tricuspid valve (TV) surgery carries an 8.8% of operative mortality [2], which is higher (12%) in the case of surgical valve replacement [3]. On the other hand, several percutaneous options are now available and under investigation [4]. In the vicious circle of the right ventricle (RV) compensation and late maladaptation to TR, RV dysfunction plays a critical role and has significant prognostic implications. The complexity is increased by the fact that the threshold for intervention in asymptomatic patients is not well defined and that symptom onset is associated with worse RV remodeling and increased operative mortality [5]. Indeed, RV dysfunction is associated with worse outcomes in both surgical [6,7] and percutaneous-treated patients [8,9]. In this light, the identification of clinical or subtle RV dysfunction before treatment is critical for patient selection and timing of intervention, especially in surgical candidates. With the development of transcatheter TV replacement (TTVR) therapies, in which the risk of afterload mismatch is potentially higher compared to transcatheter repair approaches because patients may present with more advanced disease, the assessment of RV function becomes crucial also in non-surgical candidates [10]. However, RV function evaluation is challenging, especially in the setting of altered loading conditions, as seen in severe TR, for the following reasons: (1) the increase in preload may lead to an overestimation of systolic function according to the Frank–Starling law; (2) the reliability of pulmonary artery pressure estimation from systolic right ventricular to right atrium gradient is reduced and, as a consequence, the reliability of all derived indices is decreased; (3) the particular sensitivity of RV to afterload may result in afterload mismatch after treatment that produces a reduction in preload and an increase in afterload. In this context, conventional echocardiographic indices have some limitations due to angle and load dependency, and fraction area change or ejection fraction calculation does not distinguish anterograde from retrograde flow due to regurgitation. Therefore, non-conventional echocardiographic parameters such as speckle tracking analysis or a multimodality imaging approach may be needed. The aim of this review is to provide a comprehensive evaluation of multimodality imaging of RV function assessment in severe TR. 

## 2. Anatomy and Physiology of Right Ventricle

The RV is a crescent-shaped, thin-walled structure located just behind the sternum and anterior to the left ventricle (LV). From an antero-posterior view, the RV seems to have a triangular/trapezoidal shape, while its transverse axis extends forward, wrapping around the LV [11]. The RV mass is approximately one-sixth less than LV and, in normal conditions, is adequate to guarantee a stroke volume equal to LV, mainly due to the presence of lower resistances in pulmonary vasculature [12]. Another characteristic of the RV is its wall thickness, which is thinner than the LV, ranging from 3 mm to 7 mm and up to 1.5 mm at the apex level. Based on the above, RV tolerates better volume overload than pressure overload, in contrast to LV. RV can be anatomically divided into three segments: the inflow tract, the apex and the outflow tract (right ventricle outflow tract, RVOT). The inflow tract contains the tricuspid valve and the sub-valve apparatus and extends from the tricuspid valve annulus to the insertion of papillary muscles. The apex is characterized by the accentuation of the trabeculae, which is thicker than LV. The RVOT is a tubular segment consisting of smooth and trabecular-free musculature, which supports the pulmonary valve. Three prominent muscular bands are present in the RV: the parietal band, the septomarginal band, and the moderator band [13]. The moderator band is a muscular structure that connects the anterior RV septum to the anterior papillary muscle (APM). It acts as a primary conduction path for fibers originating from the right bundle branch and directed to the free wall, but its mechanical role is to contract the APM to prevent the tricuspid regurgitation through tension of the chordae tendineae [14]. The primary function of the RV is to receive systemic venous return and to pump it into the pulmonary artery. There are two main layers of the RV myofibers: (1) a subepicardial circumferential layer, which is in continuity with the LV, and (2) a subendocardial longitudinal layer, oriented from base to apex [15]. The RV systolic function depends on four main mechanisms: longitudinal shortening, radial inward movement of the free wall (bellows effect), traction of the free wall secondary to LV contraction, and peristaltic-like motion [13]. The movements of twisting and rotation, in contrast with LV, are poorly represented and less important for RV contraction. The longitudinal contraction is due to the subendocardial longitudinal fibers, which shorten the long axis and move the tricuspid annulus towards the apex, contributing to generating approximately 75% of RV stroke volume [16]. Instead, the radial shortening is due to the contraction of the circumferential fibers in the subepicardial layer, allowing the movement of the RV free wall toward the interventricular septum (bellows effect). The third component originates from LV contraction and represents up to 40% of RV contractile force [15]. The interventricular septum contributes to the contraction of the RV through the mechanism of ventricular interdependence: the LV contraction produces a reduction in the volume of the RV due to an inward motion of the RV free wall caused both by thickening of the interventricular septum and by the contraction of fibers connecting the two ventricles [17]. In addition, the RVOT represents an anatomic separate structure that actively contributes to stroke volume with a contraction occurring with a 25–50 milliseconds delay compared to the RV body and remains contracted longer [18]. This sequential RV activation, starting with the contraction of the inlet and trabeculated myocardium and ending with the contraction of the RVOT, allows an intraventricular pressure drop of 15 mmHg and coordinates blood flow from the inflow to the outflow tract, thus generating a “peristaltic-like” motion. The RVOT function, as a resistive element, prevents the high sinus pressure from reaching the pulmonary artery and, thanks to its curvature and inotropic response, develops a helical flow that contributes to better flow stability in the pulmonary artery [19].

## 3. Multimodality Imaging in Right Ventricular Function Evaluation

Due to its peculiar anatomy and physiology of contraction, two-dimensional (2D) transthoracic echocardiography (TTE) provides a partial point of view regarding RV function and dimensions. However, in clinical practice, the RV is mainly assessed with 2D TTE, representing a rapid, non-invasive, low-cost and widely available imaging technique. However, conventional 2D parameters have limitations due to angle and load dependency and do not represent RV global function. Speckle-tracking with free wall longitudinal strain analysis provides an angle-independent and less load-dependent measurement, while three-dimensional (3D) echocardiography allows for global RV volumes and function assessment, including also RVOT. However, poor acoustic windows or difficult border delineation may reduce feasibility and reproducibility. For all the above-mentioned reasons, a comprehensive assessment of the RV requires a multimodality imaging approach, taking into account other techniques, such as cardiac magnetic resonance (CMR) and cardiac computed tomography (CCT). An overview of the main echocardiographic indices evaluating RV systolic function, with associated advantages and disadvantages, is reported in Table 1.

## 4. Echocardiography

A standardized method of RV dimensions quantification is necessary, especially aimed at reducing the inter-observer variability associated with echocardiography [20]. RV dimensions are best estimated using an RV-focused apical four-chamber view. Basal RV linear dimension (maximum transverse dimension in the basal one-third of the RV inflow at the end of diastole) and mid-cavity RV linear dimension (transverse RV diameter in the middle third of the RV inflow at the level of the papillary muscles at the end of diastole) are two of the most commonly used parameters to quantify the RV sizes because they are easy and quick to obtain [11]. However, the RV sizes depend on the angle of the probe and may be underestimated due to the characteristic crescent shape of this cardiac chamber. In general, a diameter of >41 mm at the base and >35 mm at the midlevel indicates an RV dilatation. Another commonly used parameter is the RV end-diastolic area, which is measured in the apical four-chamber view by tracing the endocardial borders of the RV at the end of the diastole. This parameter can be difficult to measure if the RV free wall is not clearly visible or the RV has many trabeculae. The RV systolic function is evaluated using several echocardiographic parameters. The following are the main indices used in clinical practice: Tricuspid Annular Plane Systolic Excursion (TAPSE), S’ of the tricuspid annulus at TDI (TDI-S’ velocity), RV Fractional Area Change (RV-FAC), thrre dimensional right ventricular ejection fraction (3D-RVEF) and RVfree wall longitudinal strain (RV-FWLS) [21]. TAPSE measures the RV longitudinal function by assessing the displacement of the tricuspid annulus towards the apex during systole. This is measured with M-mode echocardiography, aligning the cursor along the lateral tricuspid annulus from the apical 4-chamber approach. TAPSE is an easy, fast, widely available and reproducible parameter to determine. It is characterized by the following limitations: it represents only the RV longitudinal function and not the global RV systolic function; it is angle- and load-dependent; it ignores the contribution of the interventricular septum and of RVOT to the systolic phase [22]. Similarly to TAPSE, TDI-S’ velocity also reflects only the displacement of a single point of tricuspid annulus and is characterized by angle and load dependence. It is important that the lateral portion of the tricuspid annulus is aligned with the Doppler slider to avoid underestimation of measure. A value of TAPSE < 17 mm or of TDI-S’ velocity < 9.5 cm/s is highly suggestive of RV systolic dysfunction. Despite the above-mentioned limitations, TAPSE and TDI-S’ velocity resulted in an established prognostic value [23,24]. RV-FAC is an easy and widely available parameter evaluating both the RV longitudinal and radial systolic function. It is important to have a good view of both the RV apex and the RV free wall in the 4-chamber apical projection to trace the area at the end of the diastole and systole. In addition, RV-FAC presents a close correlation with RVEF CMR-derived [25]. RV-FAC is characterized by the following limitations: it is time-consuming; presents poor reproducibility; is strongly dependent on the echocardiographic imagine quality; is load dependent; and it does not include the RVOT. The ability of RV-FAC to predict major adverse cardiac events has been demonstrated in several cardiovascular diseases, including heart failure with reduced ejection fraction [26], myocardial infarction [27] and pulmonary arterial hypertension [28]. A value of RV-FAC < 35% indicates an RV systolic dysfunction. Conversely, 2D-RVEF is not recommended for clinical use due to its inaccuracy [11]. RV-FWLS is used to analyze the myocardial deformation of the RV free wall during the entire cardiac cycle. It should be measured in the apical RV-focused view, as the RV free wall is generally better visualized, and speckle tracking measurements are more reproducible [29]. It is necessary to accurately position the region of interest, limiting it to the myocardium and avoiding the pericardium and the atrial side of the tricuspid annulus. The RV is divided into six segments (at the basal, mid and apical levels), corresponding to six time–strain curves. The RV-FWLS is calculated by averaging the three regional systolic peak values along the RV free wall [30]. RV-FWLS is characterized by less angle- and load-dependency than TAPSE and TDI-S’ velocity, more reproducibility, and is less influenced by RV shape and passive heart motion. However, RV-FWLS has the following limitations: the need for good quality images and post-processing; it ignores the contribution of RVOT to the systolic phase; the presence of vendor-dependent variability of normality values range [15]. Despite these limitations, RV-FWLS revealed a higher diagnostic accuracy to predict an RVEF by CMR < 45%, using an absolute cut-off value < 17% [25]. In addition, RV-FWLS has revealed an important prognostic value, additive to other conventional echocardiographic RV parameters, in many cardiological conditions: advanced heart failure [31], heart failure with reduced ejection fraction [32], myocardial infarction [33], valvulopathies [34] and pulmonary hypertension [35]. The RV-FWLS abnormality absolute values are <20%. Three-dimensional echocardiography provides a better visualization of the RV morphology, allowing a more accurate assessment of its size and function than conventional 2D imaging [36]. Compared to 2D echocardiography, it is characterized by the acquisition of full-volume data sets, including all three components of the RV (inflow, apical portion and RVOT), as well as a detailed analysis of the size, shape, function and contraction patterns of the RV. The 3D-RVEF allows a complete assessment of the RV contraction, including the RVOT, regardless of geometric assumptions [36]. In addition, the use of automated analysis software for the calculation of 3D-RVEF allows for true results and similar volumes from both a standard four-chamber apical view and an RV-focused view [37]. The 3D-RVEF has an independent prognostic value and is potentially being used to stratify the risk of cardiac death and major adverse cardiac events [38]. In addition, 3D-RVEF has been extensively validated against RVEF by CMR [39,40]. Therefore, 3D echocardiography is recommended for evaluation of RVEF [41]. The limitations of 3D-RVEF are represented by the need for a regular cardiac rhythm and patient compliance, the limited availability, the load dependency and the use of specific analysis software. A 3D-RVEF value < 45% reflects an RV systolic dysfunction.

## 5. Cardiac Magnetic Resonance

Compared to TTE, CMR is inferior in terms of temporal resolution despite having a superior soft tissue contrast resolution. In addition, CMR is independent of acoustic windows limitation, allowing for acquiring images on potentially unlimited planes and having a non-invasive tissue characterization. CMR is an imaging technique free from ionizing radiation, and the contrast agent gadolinium-based is handier than iodinated ones. However, CMR is characterized by the following limitations: electrocardiogram (ECG) gating can be difficult in arrhythmic patients; the presence of a pacemaker implies technical challenges and visual artifacts; the use is restricted to hemodynamically stable and collaborative patients; the presence of long scan time, elevated cost and limited availability [42]. CMR actually plays a key role in the global evaluation of RV diseases and their specific etiology, representing the gold standard technique for the quantification of RV volumes and function [15]. The depiction of the complex RV anatomy in CMR is allowed by the acquisition of a multiplanar set of images, independently from thoracic anatomy and acoustic windows. Balanced Steady State Free Precession (bSSFP) images are used both to measure RV volumes and ejection fraction (EF) and for the quality assessment of segmental systolic contraction. RV studying protocols include short axis, long axis and axial series. On short-axis cine images acquired from the base through the apex of the RV, volumes and EF quantification can be performed through automated or manual detection of endocardial borders [43]. Age- and gender-specific reference values based on the peer-reviewed literature are available [44]. Standard long-axis images include a vertical RV inflow view, using a vertical plane aligned with the tricuspid valve, and an RVOT view, using a sagittal plane through the pulmonary valve [43]. Moreover, an axial stack of cine images can be acquired to analyze not only RV function but also to check for large vessel anomalies and cardiac and extra-cardiac left-to-right shunts. The detection of left-to-right shunts, together with an increased pulmonary/systemic flow ratio (Qp/Qs), through phase contrast sequences, could explain a right cardiac chamber enlargement [43] and, thus, play a central role in the evaluation of RV disease etiology. Quantification of RV myocardial global and segmental strain measurements is possible through the CMR application of feature tracking technology, but no accepted reference standards are currently available [45]. Then, in contrast to LV [11], no widespread RV segmentation scheme allows standardized evaluation of RV regional function. Nevertheless, qualitative assessment of RV regional contraction is suggested in the diagnostic algorithm of cardiomyopathies [46], and CMR allows the detection of RV akinetic, hypokinetic or micro-aneurysmal areas. In the diagnostic process of cardiomyopathies, CMR’s ability to perform non-invasive cardiac tissue characterization is essential in RV, as in LV. RV involving cardiomyopathy can hesitate in fibrosis or fat replacement of RV myocardium, detected, respectively, by T1-weightedand late gadolinium enhancement (LGE) sequences [43]. Unlike LV, thinner RV walls do not allow for discrimination of the myocardial layer, and for this reason, there are no different LGE patterns for ischemic or non-ischemic causes [47].

## 6. Cardiac Computed Tomography

ECG-gated CCT has an excellent spatial resolution, but its temporal resolution is inferior to TTE and CMR. Indeed, 3D acquisition enables the quantification of RV volumes and EF with no geometric assumption, regardless of acoustic window limitations [48]. Moreover, whereas calcification is easily recognized, the soft tissue contrast resolution is quite low, and iodinated contrast agents are needed to distinguish between the myocardium and the blood compartment. The need to use iodinated agents and radiation exposure prevents CT from being routinely used to assess cardiac chamber volumes and function. Therefore, ECG-gated CCT could be used as an alternative when CMR is contraindicated or in claustrophobic patients [42]. Adjunctive advantages include the concomitant evaluation of thoracic structures and the possibility of detecting pulmonary vascular disorders, such as pulmonary embolism. In addition, CCT can be used to plan percutaneous procedures such as tricuspid or pulmonary valve orthotopic or heterotopic replacement [49] and to evaluate tricuspid or pulmonary valve prosthesis. Finally, some parameters evaluated by CCT (as a TV annulus diameter > 29.2 mm/m^2^ and an RV end-diastolic volume > 128.8 mL/m^2^) resulted in independent predictors of RV dysfunction onset after tricuspid valve surgery [50].

## 7. Right Ventricle and Severe Tricuspid Regurgitation: A Comprehensive Assessment

**Figure 1:**
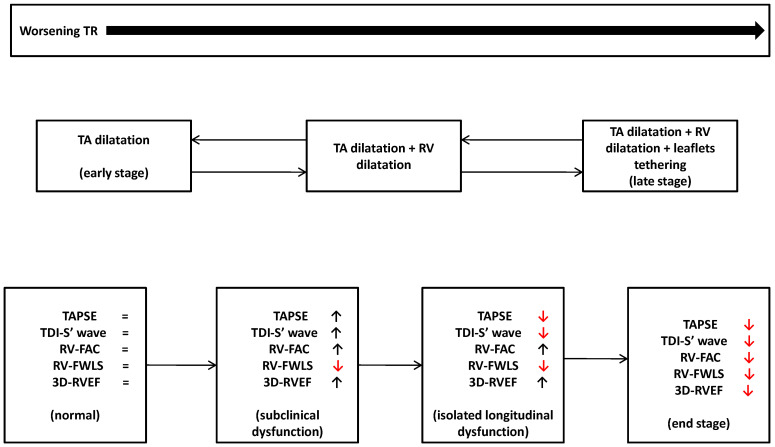
Natural history of TR. Red arrows indicate a worsening of the correspondent right ventricular echocardiographic parameter. TA: tricuspid annulus; TR: tricuspid regurgitation; RV: right ventricle; TAPSE: Tricuspid Annular Plane Systolic Excursion; TDI-S’ velocity: S’ of the tricuspid annulus at TDI; RV-FAC: RV Fractional Area Change; 3D-RVEF: three-dimensional right ventricular ejection fraction; RV-FWLS: right ventricle free wall longitudinal strain.

The assessment of RV function in the context of severe TR represents a very challenging topic. Firstly, TR is a multifaceted disease that may result from different etiologies: primary disease, secondary disease resulting from structural changes in the annulus (atrial TR) or in the ventricle (ventricular TR), and TR related to cardiac implantable electronic device (including, despite a low prevalence of about 3.6%, TR developing after implantation of leadless pacemakers [4,51]). Secondly, RV is a cardiac chamber that is highly sensitive to changes in preload and afterload. Given the spectrum of pathologies resulting in TR, it is difficult to summarize a general RV response to TR, as this depends on the underlying conditions. However, most patients with TR present both volume and pressure overload. In an early phase, TR induces dilation of the tricuspid annulus (TA), of the right atrium (RA), and, subsequently, of the RV [52], leading to leaflets tethering due to papillary muscle displacement. A vicious cycle characterized by progressive RV dilation, worsening tricuspid regurgitation and RV dysfunction develops [53]. RV dysfunction is characterized by initial longitudinal function impairment, compensated by an increase in circumferential function. A similar compensatory mechanism was evident in patients with systemic RV ventricles and chronic pressure overload [54]. At a later stage, the radial RV systolic function worsens, leading to a failure state. At this stage, RV is no longer able to compensate for the increased volume and pressure overload, leading to decreased cardiac output and worsening systemic congestion, resulting in more frequent hospitalizations and increased mortality. The above-mentioned physiopathological mechanisms in the presence of TR are summarized in Figure 1. Pressure–volume curves (PVL) help to understand the complex interplay between RV contractility, preload and afterload. Pure RV volume overload usually leads to RV dilation without altering the shape of the PVL, with contractility that remains preserved until a late stage, when a longitudinal systolic function worsening occurs. A septal diastolic flattening alters LV filling, raising pulmonary artery pressure (restriction–dilatation syndrome) [55] and the orientation of septal fibers, leading to a reduction in the LV contribution in RV contraction. In chronic pressure overload, the first adaptive response is concentric hypertrophy (homeometric adaptation) until contractility can no longer increase to match the afterload, and an eccentric “maladaptive” hypertrophy develops (heterometric adaptation) in which the Frank–Starling mechanism allows for maintaining the stroke volume with an increase in filling pressure. The lack of increase in RV contractility in response to afterload is called RV-PA uncoupling. In the PVL loop, the end-diastolic and end-systolic pressure–volume relationships are rightward displaced, and the end-diastolic pressure–volume point is characterized by higher volumes and pressures [15,56]. Given the complexity of RV compensation and maladaptation mechanisms in this context, the echocardiographic evaluation of RV function in severe TR cannot rely on a single measurement but should be based on a multiparametric echocardiographic assessment. In this light, integration with other imaging modalities, such as CMR and CT, will provide further insight.

## 8. Conventional Echocardiographic Indices

TAPSE and TDI-S’ velocity represent conventional echocardiographic indices used to evaluate the RV systolic longitudinal function [26]. The use of TAPSE and TDI-S’ velocity in the context of severe TR is characterized by the following disadvantages: load dependence; increased tricuspid annulus displacement leading to overestimation of these indices, secondary to angle-dependence; underestimation of systolic RV function after tricuspid valve annuloplasty [57]. RV-FAC is another conventional index reflecting the RV radial function, characterized by load-dependency, such as TAPSE and TDI-S’ velocity [29]. Studies evaluating the prognostic role of RV dysfunction, defined on the basis of conventional echocardiographic indices in the setting of severe TR, reported controversial results, probably due to the above-mentioned limitations of these indices. An overview of the main studies is reported in Table 2. A registry of 1298 patients with severe functional TR treated medically showed that a TAPSE value < 17 mm, regardless of the presence of RV dilatation, was associated witha reduced 5-year survival rate [58]. Dreyfus et al. developed a clinical score used to evaluate the in-hospital mortality rate in severe primary or functional TR patients who underwent isolated cardiac surgery intervention. In this study, moderate/severe RV dysfunction was assessed through TAPSE, TDI-S’ velocity and RV-FAC were among the clinical variables included in the score [59]. This evidence was confirmed in a retrospective study by Subbotina et al. [60] that enrolled a cohort of 191 patients with significant TR who underwent tricuspid valve repair/replacement intervention. The evidence of a pre-operative RV dysfunction (mean TAPSE of 13 ± 3 mm) was a predictor of early postoperative mortality and morbidity. In addition, a retrospective study by Algarni et al. [61] included 548 patients with functional TR who underwent surgical repair with concomitant left-side valve surgery, concluding that the presence of pre-operative moderate/severe RV dysfunction, defined as TAPSE < 15 mm, was an independent predictor of increased mortality. Conversely, an observational study by Karam et al. [62] included 249 patients with ≥3+ TR grade treated through transcatheter edge-to-edge valve repair. The rate of 1-year survival and survival free from heart failure (HF) hospitalization did not show any significant differences, based on evidence of pre-operative RV dysfunction defined only with TAPSE and RV-FAC. Kresoja et al. [54] evaluated the role of global and/or isolated longitudinal RV dysfunction, defined, respectively, as RVEF ≤ 45% at CMR and/or TAPSE < 17 mm, in the role of predictors of all-cause mortality or first HF hospitalization in 79 patients undergoing to transcatheter tricuspid valve repair. As a result, the evidence of global RV dysfunction (RVEF ≤ 45%), but not of an isolated longitudinal RV dysfunction, was associated with the composite endpoint. Finally, Tanaka et al. enrolled a cohort of 204 patients undergoing transcatheter tricuspid valve repair, divided on the presence of RV dysfunction, definedas RV-FAC < 35%. Three months from the intervention, an increase of RV-FAC was associated with an improved composite of all-cause mortality and HF hospitalization at 1 year in the subgroup with RV dysfunction at baseline [63,64].

## 9. Right Ventricular Free Wall Longitudinal Strain

The impairment of RV longitudinal systolic function represents an early marker of subendocardial fiber damage due to mixed volume and pressure overload in patients with severe TR [54]. The main studies that report a stronger prognostic role of RV-FWLS compared to conventional echocardiographic indices in severe TR context are summarized in Table 3. Prihadi et al. [65] enrolled 896 patients with moderate/severe functional TR, evaluating the presence of RV dysfunction, defined as TAPSE < 17 mm, FAC < 35% or RV-FWLS < 23% (absolute values). This study demonstrated that RV-FWLS was independently associated with all-cause mortality, identifying a higher rate of RV dysfunction than TAPSE and RV-FAC evaluation. A study by Ancona et al. [66] showed that RV-FWLS was able to reclassify between 42% and 56% of patients with severe TR and normal RV systolic function, defined on the basis of conventional echocardiographic parameters, to RV dysfunction. The study has also identified a cut-off of RV-FWLS absolute values < 17% and <14% as independent predictors of hospitalization for RV heart failure and of all-cause mortality, respectively. Similarly, RV-FWLS was able to better detect RV subclinical dysfunction in a study by Hinojar et al. [67], who included 151 patients with severe, massive or torrential functional TR at high risk for surgical intervention. The study showed that RV-FWLS predicted a composite endpoint of mortality and HF, independent of additive prognostic markers, thus representing a stronger predictor than conventional echocardiographic parameters. In addition, reduced RV-FWLS absolute values were associated withall-cause mortality in any grade of TR [68] and in isolated severe TR patients [69]. RV strain could also serve as a tool to select appropriate surgical candidates for TR repair at an early stage of the disease before irreversible RV damage. Indeed, in a population of 115 patients with isolated severe functional TR treated surgically, a pre-operative absolute value of RV-FWLS < 24% was associated witha composite outcome of cardiac mortality and re-hospitalization for cardiac causes within 5 years from the surgery, independent of other clinical risk factors [69]. Recently, Akintoye et al. [70] confirmed the value of RV-FWLS as discriminator to guide early intervention in asymptomatic significant TR: in a cohort of 325 patients, RV-FWLS and RV-FAC were predictors of survival with optimal threshold of <19% and a regurgitant volume > 45 mL to discriminate mortality; asymptomatic patients who met both criteria had a survival as poor as the symptomatic TR cohort with lower operative mortality, representing a group that is likely to benefit most from surgery.

## 10. Three-Dimensional Echocardiography Right Ventricular Ejection Fraction 

The 3D-RVEF is a load-dependent parameter that could potentially lead to an overestimation of RV systolic function in the context of severe TR due to volume and pressure overload and, subsequently, reduced RV stroke volume [72]. A metanalysis by Sayour et al. [73] showed the superiority of 3D-RVEF, when compared to TAPSE, TDI-S’ velocity and RV-FWLS, to predict a composite of all-cause mortality and/or adverse cardiopulmonary events, in 1928 patients with various cardiopulmonary conditions, other than severe TR. In addition, a 3D-RVEF value < 45% predicted a composite of 1-year all-cause mortality and HF hospitalization in 75 patients with severe functional TR who underwent transcatheter edge-to-edge [74]. Further, large-scale studies are needed to better evaluate the prognostic role of 3D-RVEF in patients with severe TR.

## 11. Right Ventricular–Pulmonary Arterial Coupling

RV–pulmonary arterial (PA) coupling is an index that reflects the relationship between the RV systolic function and RV afterload [75], playing a prognostic role in various cardiac conditions, such as pulmonary arterial hypertension [76], significant functional TR [77] and significant functional mitral regurgitation [78]. RV-PA coupling can be routinely evaluated non-invasively with TTE throughthe ratio between TAPSE and the estimate of pulmonary artery systolic pressure (PASP). RV-PA uncoupling, defined as TAPSE/PASP < 0.31 mm/mmHg, resulted as an independent predictor of all-cause mortality in a cohort of 1149 patients with moderate or severe secondary TR [77]. RV-PA coupling can also be evaluated with the ratio between RV-FWLS and PASP, although there are currently poor evidences in the literature. A recent study by Ancona et al. [79] revealed that, in 250 patients with severe TR, a value of RV-FWLS/PASP < 0.34%/mmHg was associated with evidence of RV failure at baseline, while a value of <0.26%/mmHg represents a better predictor of all-cause mortality than TAPSE/PASP. In massive and torrential TR, there is an inadequate estimation of RV/PA coupling due to the equalization of pressures between right heart chambers, leading to echocardiographic PASP underestimation and, subsequently, poor correlation with invasive PASP. Indeed, a study showed that TAPSE/PASP measured by right heart catheterism has a predictive role, compared to non-invasive esteemed TAPSE/PASP, in a cohort of patients with more than severe TR who underwent transcatheter tricuspid valve annuloplasty [80]. Despite these limitations in patients with more than severe TR, TAPSE/PASP was associated with major outcomes in patients undergoing transcatheter tricuspid valve repair or replacement [81,82]. Brener et al. [82] revealed that TAPSE/PASP value ≤ 0.406 mm/mmHg was associated withall-cause mortality in a cohort of 444 patients undergoing transcatheter tricuspid valve procedure. Finally, RV-PA coupling may also be estimated by the ratio between RV stroke volume and RV end-systolic volume (RV forward SV/ESV) by 3D echocardiography, avoiding the use of non-invasive PASP estimated by echocardiography [83]. Indeed, in a recent study by Gavazzoni et al. [83], a value of RV forward SV/ESV < 0.4 was found to correlate with a composite of all-cause death and HF hospitalization in patients with moderate or severe functional TR.

## 12. CMR 

RVEF CMR-derived is the gold standard measurement of RV systolic function, as it is inclusive of both longitudinal and radial RV systolic function, is very reproducible and is not based on geometric assumptions [84]. TTE constitutes the method of reference to evaluate TR severity, while CMR represents the gold standard for RV volumes and EF assessment [6]. Pre-operative RVEF by CMR resulted in being an independent predictor of cardiac death and major postoperative cardiac events in 75 patients with severe functional TR treated by TV surgery [85]. CMR allowed for identifying three different RV contraction patterns in patients with severe TR undergoing transcatheter treatment: (1) preserved longitudinal and radial function; (2) reduced longitudinal with compensatory increased radial function; and (3) reduced both longitudinal and radial function. The latter group showed the worst survival despite successful relief of RV volume overload. In this setting, an RVEF < 45% measured by CMR, in contrast to an isolated longitudinal RV dysfunction (TAPSE < 17 mm), resulted as an independent predictor of cardiac death and HF hospitalization [54]. Indeed, a reduced RVEF by CMR implies an advanced state of RV dysfunction in the natural history of TR. Therefore, RVEF CMR-derived could overestimate the RV function in severe TR, potentially not constituting an early marker of RV dysfunction [86]. Effective RVEF (eRVEF) is a novel parameter measured by CMR, characterized by less load dependency compared to RVEF: it is obtained by the ratio between the net pulmonary forward flow and RV end-diastolic volume. In a recent study by Hinojar et al. [86], the eRVEF revealed a higher proportion of RV dysfunction than RVEF by CMR and was associated with a composite of cardiovascular mortality and HF hospitalization, above RVEF. Additionally, the pressure and volume overload related to severe TR could lead to RV myocardial fibrosis, potentially detectable through LGE. However in contrast to LV, RV tissue characterization via CMR is difficult, mainly due to its thin walls [47,87]. CMR also allows for the evaluation of the RV free wall myocardial deformation and longitudinall function from routine cine images without the TTE-related disadvantages [45]. RV-FWLS by CMR resulted in being an independent predictor of all-cause mortality among 544 patients with severe functional TR, showing additive value over RVEF by CMR [88]. However, further studies are needed to establish a threshold value of RV-FWLS by CMR to be used in clinical practice with a prognostic role [45].

## 13. CCT

ECG-gated CCT guarantees the acquisition of high-quality RV images throughout the entire cardiac cycle due to its higher spatial resolution, allowing for measuring end-diastolic and end-systolic volumes, RVEF [89] and tricuspid annular dimensions [90]. In addition, RV volumes and EF obtained with CCT showed a good correlation with correspondent CMR measurements [91]. In a recent study by Tanaka et al. [92], RVEF by CCT resulted in being a predictor of all-cause mortality and HF hospitalization within 1 year after transcatheter tricuspid valve repair, providing additive information compared to TTE. However, CCT measurements of RV function in severe TR require further validation [4]. Finally, CCT plays a critical role in the pre-procedural planning of percutaneous tricuspid valve replacement, annuloplasty and caval valve intervention [93,94]. 

## 14. Use of Multimodality Imaging to Assess Rv Function

**Figure 2:**
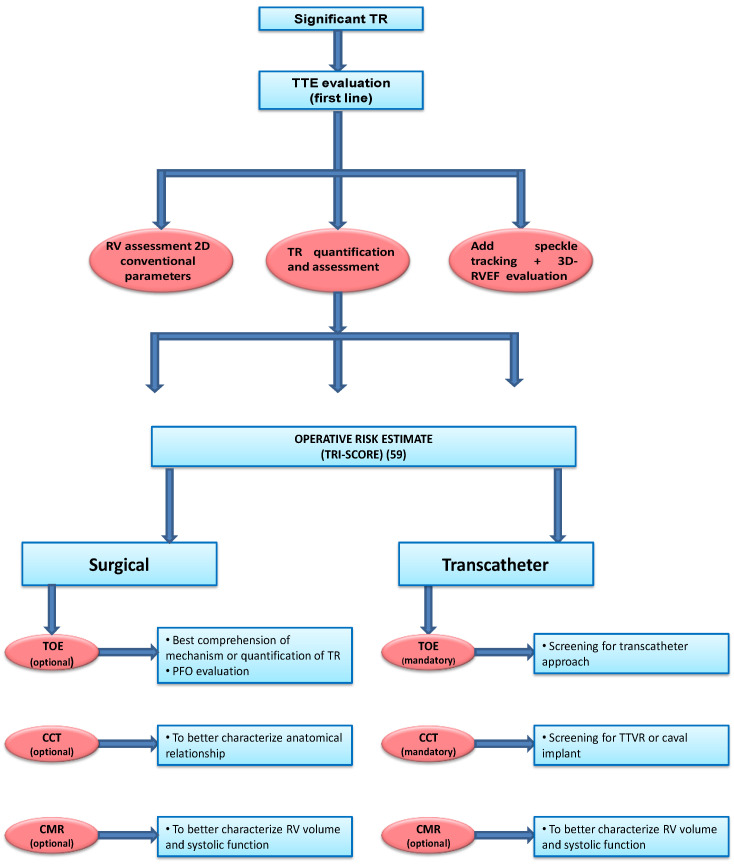
A practical approach to significant tricuspid regurgitation. TR: tricuspid regurgitation; RV: right ventricle; TTE: transthoracic echocardiography; 3D-RVEF: three-dimensional right ventricular ejection fraction; TOE: transesophageal echocardiography; CMR: cardiac magnetic resonance; CCT: cardiac computed tomography; PFO: patent foramen ovale; TTVR: transcatheter tricuspid valve replacement.

In echocardiography with conventional and non-conventional parameters, CCT and CMR both have strengths and weaknesses, so their use and combination depend on the different clinical scenarios [95]. Echocardiography represents the first imaging modality, especially in patients with severe TR, in whom TR quantification and mechanism identification are fundamental. In this setting, speckle tracking analysis and, if available, 3D echocardiography should be routinely used to account for load alterations connected to TR. CMR is the gold standard for volume and function assessment but is limited by availability, costs and the patient’s capability to perform the exam. The authors propose a personal and practical flow chart (Figure 2) for the use of multimodality imaging: if the patient may be a candidate for surgery, echocardiography with speckle tracking analysis and 3D may provide sufficient information for RV evaluation. If the imaging is suboptimal or discordant values are obtained, CMR should integrate RV dimensions and function assessment. If the patient is at high surgical risk and undergoes screening for transcatheter treatments, TTE with conventional and non-conventional parameters should be integrated with TEE valvular evaluation and with CCT for RV and annular dimension, cavo-atrial junction anatomy, etc. As mentioned previously, CMR may be integratedinto function and volume assessment.

## 15. Future Perspective

Knowledge gaps in RV assessment are still relevant and have been reviewed elsewhere [96]. Much has been conducted to improve RV global assessment thanks to speckle tracking analysis and 3D echocardiography. However, as initially performed by CMR, non-longitudinal strain analysis, like circumferential strain, may allow for studying different components of RV mechanics [54]. Three-dimensional-derived deformation indices analysis may allow for better characterizing of RV remodeling, as shown in congenital heart disease patients [97]. The same method applied to measure myocardial work for the LV may be applied to the RV, incorporating RV longitudinal strain with pulmonary pressure [98]. CMR has the advantage of characterizing myocardial tissue, but this is still unexplored in severe TR; RV tissue characterization is limited by the thin RV free wall, but the identification of myocardial fibrosis to detect subtle myocardial damage, as for the LV, would allow for early treatment intervention. Finally, only a few studies have evaluated the prognostic role of CMR and CCT in detecting RV dysfunction and further data are needed.

## 16. Conclusions

In severe TR, the identification of clinical or subtle RV dysfunction plays a critical role in patient selection and timing of surgical or transcatheter tricuspid valve intervention. Two-dimensional TTE historically represents the first-line imaging technique for RV function evaluation, but it is limited by several disadvantages. The presence of novel echocardiographic parameters and techniques, such as RV-FWLS, RV-PA coupling and 3D-RVEF, allows for earlier identification of RV dysfunction in this setting. However, the complexity of RV compensation and maladaptation mechanisms in response to severe TR and the limitations of echocardiographic parameters contribute to the difficulty of the RV function evaluation. For this reason, a multimodality imaging approach, comprehensive of CMR and CCT, is mandatory to better evaluate the RV systolic function in severe TR context.

## Figures and Tables

**Table 1:** Advantages and disadvantages of main RV systolic echocardiographic indices.

RV Function Indices	Advantages	Disadvantages
TAPSE	Easy and fast Wide availability Reproducibility Prognostic role	Angle and load dependency Reflects only RV systolic longitudinal function Excludes the contribution of interventricular septum and RVOT to systolic phase
TDI-S’ velocity	Easy and fast Wide availability Reproducibility Prognostic role	Angle and load dependency Reflects only RV systolic longitudinal function Excludes the contribution of interventricular septum and RVOT to systolic phase
RV-FAC	Easy Wide availability Reflects RV systolic radial function Correlation with RVEF-CMR derived Prognostic role	Load dependency Dependency on quality images Low reproducibility Time-consuming Excludes the contribution of RVOT to systolic phase
RV-FWLS	Less angle and load dependency Reproducibility Correlation with RVEF-CMR derived Strong prognostic role	Dependency on quality images Low availability Time-consuming Dependency of post-processing phase Dependency of vendor for reference values
3D-RVEF	Not based on geometric assumptions Strong correlation with RVEF-CMR derived Strong prognostic role	Load dependency Dependency on quality images Low availability Time-consuming Necessity of regular cardiac rhythm and patient compliance (use of multibeat acquisition modality) Dependency of post-processing phase Dependency of specific analysis software

TAPSE: Tricuspid Annular Plane Systolic Excursion; TDI-S’ velocity: S’ of the tricuspid annulus at TDI; RV-FAC: RV Fractional Area Change; 3D-RVEF: three-dimensional right ventricular ejection fraction; RV-FWLS: right ventricle free wall longitudinal strain; CMR: cardiac magnetic resonance; RVOT: right ventricle outflow tract.

**Table 2:** Overview of main studies evaluating the prognostic role of conventional echocardiographic indices.

Authors	Protocol	Patients	Target	Parameters	Results
Dietz et al. [58]	Observational registry	1298	Severe functional TR treated medically	TAPSE	- Patients with TAPSE < 17 mm had lower 5-year survival rate - Survival rate was independent from RV dilatation
Dreyfus et al. [59]	Observational registry	466	Severe primary or functional TR treated surgically	TAPSE TDI-S’ RV-FAC	- Moderate/severe RV dysfunction was an independent predictor of in-hospital mortality
Subbotina et al. [60]	Observational retrospective study	191	Significant TR treated surgically	TAPSE	- A pre-operative RV dysfunction was a predictor of early postoperative mortality and morbidity
Algarni et al. [61]	Observational retrospective study	548	Functional TR treated surgically with concomitant left-side valve surgery	TAPSE	- Patients with TAPSE < 15 mm had lower long-term survival
Karam et al. [62]	Observational study	249	Moderate/Severe TR treated with transcatheter edge-to-edge valve repair	TAPSE RV-FAC	- TAPSE and RV-FAC did not predict clinical outcomes after transcatheter edge-to-edge valve repair.
Kresoja et al. [54]	Observational study	79	Significant symptomatic TR treated with transcatheter edge-to-edge valve repair	TAPSE	- Isolated TAPSE < 17 mm was not associated withmajor outcomes
Tanaka et al. [63]	Observational retrospective study	204	Significant symptomatic TR treated with transcatheter edge-to-edge valve repair	RV-FAC	- An increase in RV-FAC after intervention is associated with improved outcomes in patients with RV dysfunction at baseline

TAPSE: Tricuspid Annular Plane Systolic Excursion; TDI-S’ velocity: S’ of the tricuspid annulus at TDI; RV-FAC: RV Fractional Area Change; TR: tricuspid regurgitation; RV: right ventricle.

**Table 3:** Overview of main studies evaluating the prognostic role of RV-FWLS.

Authors	Protocol	Patients	Target	RV-FWLS Cut-Off (Absolute Values)	Results
Prihadi et al. [65]	Observational study	896	Moderate and severe functional TR	23%	- RV-FWLS was an independent predictor of all-cause mortality - RV-FWLS identified higher rate of RV dysfunction than TAPSE and RV-FAC
Ancona et al. [66]	Observational study	250	Severe TR (mostly functional)	17% (hospitalization for RV heart failure) 14% (all-cause mortality)	- RV-FWLS reclassified 42–56% of patients with normal conventional echocardiographic parameters
Hinojar et al. [67]	Observational study	151	Severe, massive or torrential functional TR	-	- RV-FWLS was independently associated with mortality and heart failure
Bannehr et al. [68]	Observational study	1089	Mild, moderate and severe TR	18%	- RV-FWLS was an independent predictor of 2-year all-cause mortality
Wang et al. [69]	Observational study	262	Isolated severe TR	11%	- RV-FWLS was an independent predictor of all-cause mortality
Kim et al. [71]	Observational study	115	Isolated severe functional TR treated surgically	24%	- RV-FWLS predicted a composite of cardiac mortality and re-hospitalization for cardiac causes within 5 years after the surgery
Akintoye et al. [70]	Observational retrospective study	325	Moderate to severe or severe asymptomatic TR	19%	- RV-FWLS was a predicted mortality in asymptomatic patients with significant TR

RV-FWLS: RVfree wall longitudinal strain; TAPSE: Tricuspid Annular Plane Systolic Excursion; TR: tricuspid regurgitation; RV: right ventricle.
